# Physical Education Teachers’ Self-Efficacy toward Including Students with Autism in Saudi Arabia

**DOI:** 10.3390/ijerph182413197

**Published:** 2021-12-14

**Authors:** Majed M. Alhumaid

**Affiliations:** Department of Physical Education, College of Education, King Faisal University, Al-Ahsa 31982, Saudi Arabia; malhumaid@kfu.edu.sa

**Keywords:** inclusive physical education, students with disabilities, inclusion, adapted physical education

## Abstract

The inclusion of students with autism in physical education (PE) curricula has become a global trend. Although the self-efficacy of PE teachers has been identified as a major factor supporting the successful implementation of inclusive PE, limited research exists on this area in non-Western societies, especially Saudi Arabia. To address this paucity, the present study aimed (1) to determine the level of self-efficacy among PE teachers in Saudi Arabia toward including students with autism in PE classes via investigating specific independent variables; and (2) to identify the predictors of Saudi PE teachers’ self-efficacy toward including students with autism. A total of 214 Saudi PE teachers (male *n* = 188; female *n* = 26; mean age = 41.07 years) participated in this study. Self-efficacy level toward including students with autism was measured using the Arabic version of the Physical Educators’ Self-Efficacy Towards Including Students with Disabilities-Autism (PESEISD-A) instrument. Statistical analysis revealed that PE teachers had a moderate level (*M* = 4.51, *SD* = 2.04) of self-efficacy toward including students with autism in PE classes. Multiple linear regression analysis indicated that age and feelings of preparedness of the teachers were significant predictors (*β* = −0.297; *p* = 0.012; *β* = 0.178; *p* = 0.012, respectively) of their self-efficacy toward including students with autism in PE classes. The findings highlight the need for continued efforts to improve Saudi PE teachers’ level of self-efficacy and preparedness towards successfully including students with autism.

## 1. Introduction

Over the last few decades, the number of students with disabilities inclusively educated in schools has grown [1]. This is consistent with the UN’s Sustainable Development Goal 4 (SDG-4), which aims to provide high-quality education for all students, both those with and without disabilities [2]. To achieve this, many countries, including Saudi Arabia, have made amendments to their policies and legislation that relate to inclusive education [3,4].

Students with and without disabilities can achieve many benefits (e.g., enhance their learning capacity, practice social skills, and improve motivation) when educated together [5]. Haegele and Sutherland’s [6] meta-analysis of qualitative studies on how students with disabilities perceived inclusion in physical education (PE) contexts found that such students experienced both positive and negative experiences when being taught in an inclusive-based PE lesson. In particular, the authors identified that students with disabilities who tended to report positive experiences of inclusive PE classes were strongly affected by the attitude(s) of their PE teachers [6]. Therefore, PE teachers are more likely to play a critical role in the success of inclusion in PE settings.

Successfully achieving inclusion in PE depends on having well-prepared and well-qualified PE teachers [7,8]. Despite the pivotal role PE teachers play in ensuring that such inclusive educational policies are successfully implemented [9,10,11], overall, PE teachers remain under-prepared and ill-equipped to meet the challenges of providing inclusive educational environments [12]. This has reflected negatively on general PE classes, with some PE teachers reporting that they struggle to include students with disabilities in their general PE classes and often feel unable to satisfy this cohort’s specific educational needs [13,14], resulting in negative experiences of PE for both students and teachers [15,16].

Self-efficacy (SE) represents one of the most crucial elements in achieving successful pupil inclusion in general PE class contexts [17]. SE is defined as “people’s beliefs about their capability to exercise control over their own level of functioning and over events that affect their lives” [18] (p. 257). High levels of teacher SE correlate with a greater willingness to formulate novel approaches to achieve inclusion for students with disabilities [19]. Moreover, while teachers may report high levels of SE in teaching a particular subject to a particular student cohort, they may report lower levels of SE when teaching the same subject to another cohort. For instance, the majority of PE teachers report feeling capable and confident when teaching students without disabilities; however, their confidence in their abilities often falls when they are required to teach students with disabilities [20]. This trend is also common when teachers are required to teach students with different types of disabilities [21]; specifically, the specific form of student disability strongly influences teachers’ attitudes (and thus their SE) towards providing effective, inclusive PE classes [22,23]. For example, Alhumaid et al. [24] found that Saudi Arabian pre-service PE teachers reported having lower levels of SE toward including students with physical disabilities compared with their levels of SE toward teaching students with other types of disabilities, such as intellectual and visual impairments. In addition, Campos et al. [25] reported that Portuguese PE teachers face more complex difficulties when teaching students with visual impairments in inclusive PE classes compared to teaching those with intellectual and physical disabilities. Therefore, further research is needed into the level of SE among PE teachers toward including students with other types of disabilities in Saudi Arabia (e.g., students with autism).

Lindblom et al.’s [26] findings highlighted that teachers felt negative about providing inclusive classes for students with autism. Students with autism face linguistic and cognitive difficulties that impair their communication and social interaction skills, which general education teachers find challenging [27,28]. The most up-to-date reports on autistic students in the US reveal that 1 in 59 students is affected by autism, a 123% increase over the past eight years [29,30]. This has led to more students than ever with autism now being taught in inclusive schools [31]. In addition, this rise means that growing numbers of students with autism are now being taught in general PE classes [32]. This has led researchers to identify the significant concerns that general PE teachers report when they are required to provide inclusive classes for students with autism (e.g., inattention, hyperactivity, emotional difficulties, social isolation, cognitive impairments, difficulties in performing required tasks) [33]. In light of these new challenges, now more than ever, teachers must understand and be able to cater for students with autism in their inclusive classes, and have the necessary confidence in their skills and capabilities to apply these approaches in specific educational settings, especially providing inclusive PE classes for students with autism [34].

Teaching students with disabilities alongside their peers without disabilities in inclusive PE has become commonplace [35,36]. Providing inclusive PE classes for students with disabilities is a crucial part of UNESCO’s requirements [37], as inclusion facilitates the wider acceptance of students with disabilities by others [38]. However, one of the key challenges to ensuring the provision of successful inclusive PE classes is poor teacher SE [39,40]. Teacher SE towards providing inclusive PE classes for students with disabilities depends mainly on teachers’ prior experiences of teaching this cohort, their knowledge and formal training, and the type and severity of the disabilities their students have [41]. The literature indicates several factors that influence PE teachers’ SE and preparation toward the inclusion of students with disabilities. First, Shaukat et al. [42] report that teachers’ age has a significant effect on their SE toward teaching students with disabilities. Specifically, age appears to be a key factor influencing teachers’ attitudes towards adopting educational practices such as inclusive education [1]. In terms of gender, Fournidou et al. [43] reported that, in comparison to their female counterparts, male PE teachers were generally more willing to engage in inclusive educational practices to cater for students with physical disabilities. However, female teachers showed more belief in their efficacy in teaching students with disabilities than male teachers [44]. Despite this, Loreman et al. [45] found no significant differences between male and female teachers’ SE in inclusive teaching settings. Furthermore, the level of teachers’ education appeared to significantly determine SE toward including students with disabilities. For example, teachers with post-graduate degrees reported having higher levels of confidence towards teaching students with disabilities than graduates [46].

A study of nine experienced high-school PE teachers reported that they felt poorly prepared and had inadequate support and resources to successfully provide inclusive classes for students with disabilities [47]. As mentioned above, having previous experience of teaching students with disabilities also contributes significantly to teachers’ SE toward including students with disabilities [48]. For example, Alharbi et al. [49] reported that teachers with previous experience of teaching students with autism have a statistically greater level of awareness of how to overcome the challenges that teaching this cohort involves. However, Ruble et al. [50] indicated that no association between experience and SE was reported. Hutzler et al. [39] demonstrated that teachers’ experiences with relatives with a disability can influence teachers’ SE toward inclusion. Malinen et al. [51] investigated the role of prior experience of teaching students with autism among three groups of in-service teachers from Finland, China, and South Africa. The results suggest that having prior experience of teaching students with disabilities was the best predictor of teachers’ SE in achieving inclusive education goals. Additionally, while Finnish teacher training on inclusive practices significantly increased this cohort’s level of SE towards teaching students with disabilities [51], prior academic preparation was identified as the most significant predictor of favorable attitudes towards inclusion among PE teachers [52].

Although a plethora of research exists on how teacher gender, years of experience, and other independent variables affect PE teachers’ SE and preparation globally, the investigation of these factors on Saudi PE teachers’ SE toward including students with autism is very limited. Additionally, there is an evident lack of research on the levels of SE among PE teachers towards including students with autism. Therefore, to address this paucity, the present study aims (1) to determine the levels of SE toward including students with autism among Saudi PE teachers based on independent variables (e.g., age, gender, and relatives with autism); and (2) to identify the predictors of Saudi PE teachers’ SE toward including students with autism in PE classes. The researcher hopes that this study will provide baseline data on SE levels among Saudi Arabian PE teachers toward the inclusion of students with autism, in addition to providing a useful blueprint for enhancing inclusive PE practices in other non-Western countries more widely.

## 2. Materials and Methods

### 2.1. Participants

Saudi Arabian PE teachers (*N* = 214; male: *n* = 188; female: *n* = 26) aged 21–59 (*M* = 41.07 years; *SD* = 9.30 years) teaching in 13 regions of Saudi Arabia (Eastern: 44.9%; Riyadh: 18.2%; Makkah: 17.3%; Jazan: 6.1%; Asir: 3.7%; Qasim: 3.3%; Madina: 2.3%; Tabuk: 1.9%; others: 2.4%) completed an Arabic version of the Physical Educators’ Self-Efficacy Towards Including Students with Disabilities-Autism (PESEISD-A) instrument [53].

### 2.2. Measures

#### 2.2.1. Demographic Data

The demographic form was used to gather age, gender, academic degree in PE, current grade of PE class taught, school location, years of teaching experience, having a family member with autism, previous experience of teaching students with autism, attending a training course related to teaching students with autism, and feelings of preparedness for including students with autism.

#### 2.2.2. Physical Education Teachers’ Self-Efficacy

The physical education teachers’ SE was measured using the Physical Educators’ Self-Efficacy Towards Including Students with Disabilities-Autism (PESEISD-A) instrument [53]. The instrument targets ten specific areas of teacher SE: (i) how confident PE teachers feel at teaching students with autism (e.g., I am confident in my ability to modify instructions, tasks, equipment, and activities for students with autism); (ii) how confident PE teachers feel about creating a safe environment for students with autism; (iii) how confident PE teachers feel about creating and maintaining good relationships with their peers; and (iv) how confident PE teachers feel to motivate students with autism in inclusive PE contexts. The scoring system for all ten items ranges from 0 (do not feel able at all) to 10 (feel highly able). A higher score indicates a higher level of SE toward providing inclusive PE classes for students with autism. The PESEISD-A was translated from English into Arabic using the bilingual method [54] by four (Arabic and English) bilingual special education and PE professors. The translation was on the basis of the meaning of English statements rather than verbatim. No items were removed during the translation processes. The English and Arabic versions were then compared for accuracy and modifications made as required. The construct validity of the Arabic version was assessed using exploratory factor analysis and principal component analysis, and its reliability was assessed via Cronbach’s alpha.

#### 2.2.3. Reliability and Validity

The exploratory factor analysis and principal component results indicate that the Kaiser–Meyer–Olkin measure of sampling adequacy was 0.89, which is above the acceptable threshold of 0.6; Bartlett’s test of sphericity score was statistically significant (*p* < 0.001) [55]. This indicated that all the communalities were greater than 0.30. All item scores then underwent exploratory factor analysis; this identified that a single component related to the Arabic version of the PESEISD-A instrument accounted for 52.57% of the total variance, which confirmed that all ten items represented a unidimensional construct. The factor loading for all ten items (>0.50: 0.61–0.81) indicated that all ten items in the domain made a significant contribution. Next, the reliability of the Arabic version of the PESEISD-A was examined using Cronbach’s alpha; the results indicate good internal consistency (0.89) [56]. Additionally, all items achieved an acceptable corrected item-total correlation of >0.30 [57]. Hence, the Arabic version of the PESEISD-A instrument was reliable and valid to measure Saudi Arabian PE teachers’ levels of SE toward including students with autism.

### 2.3. Procedure

Participants were invited to join the study voluntarily. The questionnaire was delivered via email containing a link to an online questionnaire platform (Google Forms). The link led the participants to an information sheet that detailed the study’s aims and participant instructions; clicking on the questionnaire gave their implied informed consent to participate. At the end of the data collection period, the completed questionnaires (*N* = 265) were downloaded and underwent error checking to ensure the data were complete. After removing the incomplete questionnaires (*n* = 51), the fully completed questionnaires (*n* = 214) were analyzed. The protocol was approved by the Research Ethics Committee at King Faisal University, Saudi Arabia (KFU-REC/2021-08-05).

### 2.4. Data Analysis

To address the first research objective, the mean (*M*) and standard deviation (*SD*) were computed for the PESEISD-A instrument items. All distributions were checked for normality using the Shapiro–Wilk test. A nonparametric Mann–Whitney U test and a Kruskal–Wallis test were used to perform all comparisons. After checking that the assumptions had been met, a multiple linear regression (MLR) was used to address the second research objective. MLR scores were calculated to predict the level of SE among PE teachers based on the independent variables (e.g., age; relatives with autism). The Statistical Package for the Social Sciences (SPSS) (IBM SPSS Statistics 26.lnk, Chicago, IL, USA) was used to perform the statistical analysis. The significance level was set at *p* < 0.05.

## 3. Results

### 3.1. Demographic Characteristics

The participant sample consisted of 214 Saudi PE teachers aged 21–59 (*M* = 41.07 years; *SD* = 9.30 years) teaching at Saudi primary, middle, and high schools (57.5%, 26.2%, and 16.4%, respectively). Female teachers accounted for over 12% of the total participants. Most participants (78%) held a bachelor’s degree in PE, while only 9% held a PhD in PE. 96 (44.9%) of the participants were from the Eastern Region of Saudi Arabia. Table 1 provides additional relevant information on the participants’ background and SE scores.

### 3.2. The Level of Physical Education Teachers’ Self-Efficacy

Table 1 shows data related to the PE teachers’ SE towards including students with autism in PE classes. The results indicate that SE level among the participants was moderate overall (*M* = 4.51; *SD* = 2.04). In particular, the majority (52.3%) had a moderate level of SE toward including students with autism; 37.9% had a low level of SE toward including students with autism; and 10% of PE teachers had a high SE level. Significant differences between the research groups were only found for three independent variables: gender, degree, and feelings of preparedness toward inclusion; *p* = 0.032; *p* = 0.043; and *p* ≤ 0.001, respectively.

### 3.3. Predictors of Self-Efficacy

The MLR analysis showed that a significant regression model was identified (F (9204) = 2.687, *p* < 0.006), featuring an *R*^2^ score of 0.106. Therefore, the regression analysis results indicate that the independent variables accounted for 10.6% of the variation in the SE of the participants. The regression analysis indicated that no collinearity was present in the results, which suggests that the results have adequate statistical significance. Table 2 illustrates the significance, direction, and strength of the relationships among the individual predictors and the SE of the participants toward including students with autism in PE classes. Age (*β* = −0.297; *p* = 0.012), as the first predictor that achieved negative standardized beta values, suggests that older PE teachers likely have lower levels of SE toward including students with autism. Feelings of preparedness toward inclusion (*β* = 0.178; *p* = 0.012), as the second predictor with positive beta values, suggests that more well-prepared PE teachers have higher levels of SE. Age represented the strongest unique contributor to the SE of the participants (*β* = −0.297).

## 4. Discussion

The present study aimed (1) to determine the level of SE toward including students with autism among PE teachers in Saudi Arabia via investigating specific independent variables; and (2) to identify the predictors of SE toward including students with autism among Saudi PE teachers.

For the first objective, the findings reveal a moderate level of SE among the participants toward including students with autism in PE classes. Significant gender differences were evident: male Saudi PE teachers expressed higher levels of SE toward including students with autism than their female counterparts. In line with this finding, the results of Fournidou et al.’s [43] study suggest that male PE teachers had a higher overall willingness towards providing inclusive classes for students with disabilities in general PE classes compared to female PE teachers. That said, in this regard the present study’s findings contrasted with the results of other researchers [58,59,60], who found insignificant differences between the SE of male and female PE teachers towards inclusive PE practices. Similarly, Aldabas’ [61] study found insignificant differences between the levels of confidence of male and female teachers towards providing inclusive classes for students with disabilities. One potential reason that male Saudi PE teachers may report higher SE levels towards engaging in inclusive practices with students with autism is that female PE teachers likely have less experience of teaching inclusive PE classes. In support, Alahmadi [62] reports that only in 2018 did girls’ schools in Saudi Arabia begin to provide PE classes due to Saudi Arabia’s strict rules on same-sex teacher and pupil classes; this means that female Saudi PE teachers likely only have a maximum of around three years’ experience of teaching PE, let alone offering inclusive PE instruction for students with disabilities. Consequently, more research is required on this issue to control for differences between male and female Saudi PE teachers in terms of their length of experience of teaching PE inclusively.

For level of education, the results indicate that significant differences were evident between the participants: those with a master’s degree reported higher levels of SE toward including students with autism in PE classes than their counterparts with lower educational degrees (e.g., diploma). This finding concurs with Ruppar et al. [46] finding: teachers with post-graduate degrees reported feeling more confident about teaching students with disabilities than those with graduate degrees. In addition, this finding is somewhat in line with Aldabas’ [61] results; namely, that teachers with postgraduate and graduate degrees reported higher levels of confidence towards engaging in inclusive teaching practices than their counterparts with lower qualifications. One possible explanation for this finding might be that those with postgraduate degrees have amassed more knowledge and practical training on facilitating inclusive educational practices [58].

Another important finding of the present study was the significant differences between the participants’ reported feelings of preparedness towards including students with autism in their PE classes; approximately 50% of the participants reported that their academic studies had not adequately prepared them for providing inclusive PE instruction for students with autism. Several previous studies [4,63,64,65] found that undergraduate pre-service PE teacher training programs fail to provide adequate inclusivity training for pre-service PE teachers. Therefore, the above findings may explain the finding of the present study that around 50% of the participants reported that their academic studies did not adequately prepare them for providing inclusive PE instruction for students with autism.

The results on the predictors of PE teachers’ SE toward including students with autism in PE settings suggest that the independent variables age and feelings of preparedness were significant predictors. The first predictor, age, negatively predicted Saudi PE teachers’ SE toward including students with autism in PE settings; older PE teachers reported lower levels of SE toward including students with autism, while the opposite was true of younger PE teachers. In support, Özer et al.’s [66] study identified that younger PE teachers reported feeling more comfortable with teaching students with disabilities. In addition, Hwang and Evans’s [67] study on South Korean teachers found that older teachers had more favorable attitudes towards engaging in inclusive educational practices. Additionally, Schmidt and Vrhovnik’s [68] study on Slovenian PE teachers found that those in their twenties reported feeling more positive towards engaging in inclusive educational practices than those aged 30 and over. That said, the findings of other researchers contradict these results; for example, Tripp and Rizzo [69] and more recently You et al. [1] found that teacher age was not a significant factor in reporting favorable attitudes and high levels of SE toward engaging in inclusive practices. However, the present study’s finding that younger PE teachers are more likely to have higher levels of SE towards offering inclusive PE instruction may, in part, be due to a 2015 UNESCO ruling that provided students with disabilities the right to access inclusive, adapted, and safe PE classes [70]. After 2015, significant improvements to inclusive PE policies were implemented in Saudi Arabia [3]. To explain, younger PE teachers appear to have derived more benefit from these legislative changes in support of inclusive education than older PE teachers. This is likely because younger PE teachers have been exposed to the new inclusive-education-focused curricula in various countries [39], as well as having gained a greater degree of professional experience at offering inclusive PE classes.

Feelings of preparedness toward inclusion, the second significant predictor of PE teachers’ SE toward including students with autism in PE settings, was a positive predictor of teacher SE. Specifically, participants who reported feeling that their academic studies had adequately prepared them for teaching students with autism in inclusive PE classes were more likely to have high levels of SE. These findings are consistent with other researchers [20,71,72], who also concluded that the undergraduate academic courses PE teachers had taken significantly enhanced their levels of SE towards the inclusion of students with disabilities in PE settings. Aldabas [61] indicated that the majority of Saudi Arabian teachers reported feeling confident that they were prepared for teaching students with disabilities in inclusive settings. That said, the findings of Coates [73] and Hersman and Hodge [74] contradict this assertion: here, teachers commonly reported having negative attitudes and low levels of SE towards teaching students with disabilities in PE classes due to inadequate training and education, support, and teaching experience. Furthermore, as highlighted by Corona et al. [75], there is an urgent need for continued efforts to improve the quality of education and training for teachers working with students with autism. It has long been suggested that PE teachers have limited knowledge and are poorly prepared for teaching students with disabilities [13,52,66]. However, Alhumaid et al. [76] concluded that adapted physical activity training programs significantly enhance the SE levels of pre-service PE teachers toward teaching students with disabilities; this highlights the urgent need to improve the quality of pre-service educational provision for PE teachers working with students with disabilities [4].

## 5. Conclusions

Alongside the growing trend in students with autism participating in inclusive PE in schools globally [31], the present study’s findings highlight that Saudi Arabian PE teachers report having only moderate levels of SE towards providing inclusive PE classes for students with autism. This demonstrates the need for greater efforts by Saudi educational institutions and decision makers in government to ensure the successful delivery of effective inclusive PE for students with autism. The present study’s results reveal that academic preparation plays a crucial role in raising the levels of SE among Saudi Arabian PE teachers and ensuring the success of inclusive PE practices. In other words, PE teachers who have a good level of academic preparation are more likely to report higher levels of SE toward the inclusion of students with autism. Therefore, to improve SE levels and preparedness among Saudi Arabian PE teachers’ towards including students with autism, it is recommended that Saudi Arabian universities offering pre-service PE teacher training programs improve their standards in relation to improving teachers’ knowledge and experience of providing inclusive PE education for students with autism and offer more comprehensive opportunities for practicum.

## 6. Limitations and Implications

Despite its merits, the present study is subject to three main limitations (and their respective implications) that need to be addressed by future research. First, the male and female sample sizes were unequal (male: *n* = 188; female: *n* = 26) as only in 2018 did the Saudi government enact new policies allowing PE to be taught in girls’ schools. Therefore, further research featuring larger cohorts of female Saudi PE teachers is required to increase our understanding of their experiences and attitudes towards teaching students with autism and better prepare future female Saudi PE teachers for teaching in inclusive contexts. Second, due to Saudi Arabia’s size, encompassing 13 culturally diverse regions, the present study was unable to achieve an accurate comparison among PE teachers across the regions, which likely affects the generalizability of the results. To address this, it is recommended that future researchers should recruit equal numbers of male and female PE teacher participants from each of Saudi Arabia’s 13 regions. Third, because the present study gathered self-reported data from the participants via the PESEISD-A instrument, it failed to collect empirical data on the participants’ behavior in their PE teaching environments. This means that the present study findings may have been subject to participant bias [77] whereby they may have provided overly positive accounts of their SE and abilities towards providing inclusive learning for students with autism in their PE classes. Therefore, it is recommended that future researchers should apply empirically based research methods (e.g., class observations) to better understand PE teachers’ in-class behaviors towards including students with autism.

## 7. Practical Applications

The study’s findings provide baseline data on SE levels among Saudi Arabian PE teachers toward the inclusion of students with autism in PE classes. It is hoped that these findings will help Saudi educational institutions and decision makers in government to address the shortcomings of the current curricula and methodologies and develop them to ensure the successful delivery of effective, inclusive PE for students with autism. First, the findings illustrate that specific methodologies within the curriculum (e.g., providing practical experiences and more opportunities for working with students with autism) should be adopted by PE teacher education programs, given the potent effects of enhancing PE teachers’ SE on their teaching abilities and consequently on the success of inclusive practices. Second, developing programs that feature interventions such as in-service training, workshops, refresher courses, and learning materials that are more explicitly targeted towards teaching students with autism would further enhance teachers’ SE abilities to provide successful inclusive teaching. Third, more effective and appropriate educational programs could be implemented to improve inclusive teaching education among PE teachers, especially for those teaching students with autism. Finally, Saudi educational institutions and decision makers could also formulate relevant strategies and policies that will benefit both teachers and students, by developing evidence-based and inclusion-positive educational interventions to allow pre-service PE teachers to be better prepared for including students with autism in their classes.

## Figures and Tables

**Table 1:** Exploring the level of self-efficacy scores for independent variables (*N* = 214).

Independent Variables	*n*	*M ± SD*	χ^2^/z	*p* Value
Gender				
Male	188	4.63 ± 1.97	−2.143 ^a^	0.032 *
Female	26	3.65 ± 2.40		
Degree				
Diploma	21	3.71 ± 2.42	9.832 ^b^	0.043 *
Bachelor	167	4.50 ± 1.99		
High Diploma	7	4.65 ± 2.30		
Master	12	5.70 ± 1.52		
PhD	2	3.30 ± 2.70		
Teaching grade				
Primary	123	4.40 ± 2.13	0.520 ^b^	0.771
Middle	56	4.67 ± 2.04		
High	35	4.66 ± 1.74		
Teaching experience				
0–5	55	4.51 ± 2.03	0.404 ^b^	0.982
6–10	25	4.26 ± 2.11		
11–15	27	4.47 ± 2.02		
16–20	29	4.63 ± 2.07		
+20	78	4.57 ± 2.08		
Family member with autism				
Yes	21	4.60 ± 2.16	−0.145 ^a^	0.885
No	193	4.50 ± 2.04		
Previous teaching autism				
Yes	67	4.64 ± 2.30	−0.635 ^a^	0.525
No	147	4.45 ± 1.92		
Training in teaching autism				
Yes, theoretical	38	4.70 ± 2.41	5.496 ^b^	0.139
Yes, practical	2	7.45 ± 1.20		
Yes, both	15	4.76 ± 2.26		
No	159	4.41 ± 1.92		
Feelings of preparedness				
Never	101	3.90 ± 2.10	20.489 ^b^	<0.001 *
Little	94	5.23 ± 1.70		
A lot	19	4.24 ± 2.32		

Note: ^a^ Mann–Whitney U Test; ^b^ Kruskal–Wallis Test; * the mean difference is significant at the 0.05 level.

**Table 2:** Multiple linear regression analysis to predict physical education teachers’ self-efficacy.

Predictor	Unstandardised Coefficients		Standardised Coefficients			
	B	Std. Error	Beta	*t*	*p*	Partial *R*
(Constant)	4.627	1.578	-	2.933	0.004	-
Age	−0.061	0.024	−0.297	−2.541	0.012 *	−0.175
Gender	−0.155	0.539	−0.025	−0.288	0.774	−0.020
Degree	0.360	0.197	0.127	1.829	0.069	0.127
Teaching grade	0.106	0.187	0.039	0.565	0.572	0.040
Teaching experience	0.315	0.161	0.252	1.948	0.053	0.135
Family member with autism	−0.072	0.465	−0.011	−0.156	0.876	−0.011
Previous teaching autism	−0.048	0.338	−0.011	−0.142	0.888	−0.010
Training in teaching autism	−0.021	0.138	−0.012	−0.152	0.879	−0.011
Feelings of preparedness	0.565	0.223	0.178	2.538	0.012 *	0.175

* The mean difference is significant at the 0.05 level.

## Data Availability

The data that support the findings of this study are available from the author, upon reasonable request.

## References

[1] You S., Kim E.K., Shin K. (2019). Teachers’ Belief and Efficacy toward Inclusive Education in Early Childhood Settings in Korea. Sustainability.

[2] UN SDGs Transforming Our World: The 2030 Agenda for Sustainable Development. Resolution Adopted by the UN General Assembly. https://sustainabledevelopment.un.org/post2015/transformingourworld.

[3] Alharbi A., Madhesh A. (2018). Inclusive Education and Policy in Saudi Arabia. Int. J. Educ. Res. Rev..

[4] Granell J.C., Goig R.L., Raga M.G., Maher A. (2021). Perceived Competence to Teach Students with Special Educational Needs in Physical Education: The Voice of University Students from Spain and United Kingdom. Retos. Nuevas. Tendencias. Educ. Fis. Deporte. Rec..

[5] Oliver K.L., Oesterreich H.A. (2013). Student-centred Inquiry as Curriculum as a Model for Field-based Teacher Education. J. Curric. Stud..

[6] Haegele J.A., Sutherland S. (2015). Perspectives of Students with Disabilities toward Physical Education: A Qualitative Inquiry Review. Quest.

[7] Hopkins S.L., Round P.N., Barley K.D. (2018). Preparing Beginning Teachers for Inclusion: Designing and Assessing Supplementary Fieldwork Experiences. Teach. Teach..

[8] UNESCO (2017). A Guide for Ensuring Inclusion and Equity in Education.

[9] Bastos T., Teixeira J., Amaral M., Corredeira R., Alexandre J. (2017). Physical Education and Sport as a Means to Empower Children with Disability in Educational and Community Settings: The Contribution of Paralympic Education Focusing on Peers’ Interactions. Inclusive Physical Activities: International Perspectives.

[10] Haegele J.A., Wilson W.J., Zhu X., Bueche J.J., Brady E., Li C. (2020). Barriers and Facilitators to Inclusion in Integrated Physical Education: Adapted Physical Educators’ Perspectives. Eur. Phys. Educ. Rev..

[11] Kristén L., Klingvall B., Ring M. (2020). The co-development of Inclusive Tools in Physical Education for Pupils with and without Disabilities. Sport. Soc..

[12] Healy S., Block M.E., Kelly L. (2020). The Impact of Online Professional Development on Physical Educators’ Knowledge and Implementation of Peer Tutoring. Int. J. Disabil. Dev. Educ..

[13] Kwon E., Block M. (2017). Implementing the Adapted Physical Education E-learning Program into Physical Education Teacher Education Program. Res. Dev. Disabil..

[14] Neville R.D., Makopoulou K., Hopkins W.G. (2019). Effect of an Inclusive Physical Education (IPE) Training Workshop on Trainee Teachers’ Self-Efficacy. Res. Q. Exerc. Sport..

[15] Chatzipanteli A., Dean R. (2020). Teaching Styles and the Inclusion of Students with Difficulties in Regular Physical Education. J. Phys. Educ. Recreat. Dance..

[16] Tindall D., Culhane M., Foley J. (2016). Pre-service Teachers’ Self-Efficacy towards Children with Disabilities: An Irish Perspective. Eur. J. Adapt. Phys. Activ..

[17] Foley J.T., Santarossa S., Tindall D.W., Lieberman L.J. (2020). The Impact of a Summer Sports Camp for Children with Visual Impairments on the Self-Efficacy of Physical Education Pre-service Teachers: A Pilot Study. Eur. J. Adapt. Phys. Act..

[18] Bandura A. (1977). Self-efficacy: Toward a Unifying Theory of Behavioral Change. Psychol. Rev..

[19] Block M.E., Hutzler Y., Barak S., Klavina A. (2013). Creation and Validation of the Self-Efficacy Instrument for Physical Education Teacher Education Majors toward Inclusion. Adapt. Phys. Act. Q..

[20] Wang Y.-S., Liu L., Wei X.-W., Block M.E. (2020). The Self-Efficacy of Preservice Physical Education Teachers in Disabilities Education in China. Sustainability.

[21] De Boer A.A., Pijl S.J., Minnaert A. (2011). Regular Primary Schoolteachers’ Attitudes towards Inclusive Education: A Review of the Literature. Int. J. Incl. Educ..

[22] Avramidis E., Norwich B. (2002). Teachers’ Attitudes towards Integration/Inclusion: A Review of the Literature. Eur. J. Spec. Needs Edu..

[23] Schwab S. (2018). Attitudes Towards Inclusive Schooling: A Study on Students’, Teachers’ and Parents’ Attitudes.

[24] Alhumaid M.M., Khoo S., Bastos T. (2020). Self-Efficacy of Pre-service Physical Education Teachers toward Inclusion in Saudi Arabia. Sustainability.

[25] Campos M.J., Ferreira J.P., Block M.E. (2015). Exploring Teachers’ Voices about Inclusion in Physical Education: A Qualitative Analysis with Young Elementary and Middle School Teachers. Compr. Psychol..

[26] Lindblom A., Dindar K., Soan S., Kärnä E., Roos C., Carew M.T. (2020). Predictors and Mediators of European Student Teacher Attitudes toward Autism Spectrum Disorder. Teach. Teach. Educ..

[27] Alshaigi K., Albraheem R., Alsaleem K., Zakaria M., Jobeir A., Aldhalaan H. (2020). Stigmatization among Parents of Autism Spectrum Disorder Children in Riyadh, Saudi Arabia. Int. J. Pediatr. Adolesc. Med..

[28] Beamer J.A., Yun J. (2014). Physical Educators’ Beliefs and Self-Reported Behaviors toward Including Students with Autism Spectrum Disorder. Adapt. Phys. Act. Q..

[29] Baio J., Wiggins L., Christensen D.L., Maenner M.J., Daniels J., Warren Z., Kurzius-Spencer M., Zahorodny W., Rosenberg C.R., White T. (2018). Prevalence of Autism Spectrum Disorder among Children aged 8 years—Autism and Developmental Disabilities Monitoring Network, 11 sites, United States, 2014. MMWR. Surveill. Summ..

[30] Maenner M.J., Shaw K.A., Baio J. (2020). Prevalence of Autism Spectrum Disorder among Children aged 8 years—Autism and Developmental Disabilities Monitoring Network, 11 sites, United States, 2016. MMWR. Surveill. Summ..

[31] Lu M., Zou Y., Chen X., Chen J., He W., Pang F. (2020). Knowledge, Attitude and Professional Self-Efficacy of Chinese Mainstream Primary School Teachers Regarding Children with Autism Spectrum Disorder. Res. Autism Spectr. Disord..

[32] Li C., Wang L., Block M.E., Sum R.K., Wu Y. (2018). Psychometric Properties of the Physical Educators’ Self-Efficacy Toward Including Students with Disabilities—Autism among Chinese Preservice Physical Education Teachers. Adapt. Phys. Activ. Q..

[33] Obrusnikova I., Dillon S.R. (2011). Challenging Situations when Teaching Children with Autism Spectrum Disorders in General Physical Education. Adapt. Phys. Activ. Q..

[34] Bandura A. (1997). Self-Efficacy: The Exercise of Control.

[35] Heck S., Block M.E. (2019). Inclusive Physical Education around the World: Origins, Cultures, & Practices.

[36] Pellerin S., Wilson W.J., Haegele J.A. (2020). The Experiences of Students with Disabilities in Self-Contained Physical Education. Sport Educ. Soc..

[37] UNESCO (2008). Inclusive Education: The Way of the Future. Proceedings of the International Conference on Education 48th Session.

[38] Block M.E., Grenier M., Hutzler Y., Alexandre J.S., Christophe M., Danielle T., Rhonda G. (2017). Strategies to Maximize Social Participation and Inclusion of Students with Disabilities in Physical Education. Inclusive Physical Activities: International Perspectives.

[39] Hutzler Y., Meier S., Reuker S., Zitomer M. (2019). Attitudes and Self-Efficacy of Physical Education Teachers toward Inclusion of Children with Disabilities: A Narrative Review of International Literature. Phys. Educ. Sport. Pedagog..

[40] Reina R., Healy S., Roldan A., Hemmelmayr I., Klavina A. (2019). Incluye-T: A Professional Development Program to Increase the Self-Efficacy of Physical Educators towards Inclusion. Phys. Educ. Sport. Pedagog..

[41] Casebolt K.M., Hodge S.R. (2010). High School Physical Education Teachers’ Belief about Teaching Students with Mild to Severe Disabilities. Phys. Educ..

[42] Shaukat S., Vishnumolakala V.R., Al Bustami G. (2019). The Impact of Teachers’ Characteristics on their Self-Efficacy and Job Satisfaction: A Perspective from Teachers Engaging Students with Disabilities. J. Res. Spec. Educ. Needs..

[43] Fournidou I., Kudlacek M., Evagellinou C. (2011). Attitudes of In-Service Physical Educators toward Teaching Children with Physical Disabilities in General Physical Education Classes in Cyprus. Eur. J. Adapt. Phys. Act..

[44] Shaukat S., Sharma U., Furlonger B. (2013). Pakistani and Australian Pre-Service Teachers’ Attitudes and Self-Efficacy towards Inclusive Education. J. Behav Sci..

[45] Loreman T., Sharma U., Forlin C. (2013). Do Pre-Service Teachers Feel Ready to Teach in Inclusive Classrooms? A Four Country Study of Teaching Self-Efficacy. Aust. J. Teach. Educ..

[46] Ruppar A.L., Neeper L.S., Dalsen J. (2016). Special Education Teachers’ Perceptions of Preparedness to Teach Students with Severe Disabilities. Res. Pract. Pers. Sev. Disabil..

[47] Hodge S., Ammah J.O., Casebolt K.M., Lamaster K., Hersman B., Samalot-Rivera A., Sato T. (2009). A Diversity of Voices: Physical Education Teachers’ Beliefs about Inclusion and Teaching Students with Disabilities. Int. J. Disabil. Dev. Educ..

[48] Hutzler Y., Barak S. (2017). Self-Efficacy of Physical Education Teachers in Including Students with Cerebral Palsy in their Classes. Res. Dev. Disabil..

[49] Alharbi K.A., Alharbi A.A., Al-Thunayyan F.S., Alsuhaibani K.A., Alsalameh N.S., Alhomaid M.H., Albahouth I.S., Hamid P.F. (2019). School’s Teachers Knowledge about Autism in Al-Badayacity, Al-Qassim Region, Kingdom of Saudi Arabia. Mater. Socio-Med..

[50] Ruble L.A., Usher E.L., McGrew J.H. (2011). Preliminary Investigation of the Sources of Self-Efficacy among Teachers of Students with Autism. Focus Autism Other Dev. Disabil..

[51] Malinen O.P., Savolainen H., Engelbrecht P., Xu J., Nel M., Nel N., Tlale D. (2013). Exploring Teacher Self-Efficacy for Inclusive Practices in three Diverse Countries. Teach. Teach. Educ..

[52] Hodge S.R., Elliott G. (2013). Physical Education Majors’ Judgments about Inclusion and Teaching Students with Disabilities. J. Educ. Trng. Stud..

[53] Taliaferro A., Block M., Harris N., Krause J. (2010). Physical Educators’ Self-Efficacy toward Including Students with Disabilities-Autism Version 8.2.

[54] Brislin R.W. (1970). Back-Translation for Cross-Cultural Research. J. Cross-Cult. Psychol..

[55] Tabachnick B.G., Fidell L.S. (2001). Using Multivariate Statistics.

[56] Nunnally J. (1978). Psychometric Theory.

[57] Cristobal E., Flavian C., Guinaliu M. (2007). Perceived E-service Quality (PeSQ): Measurement Validation and Effects on Consumer Satisfaction and Web Site Loyalty. Manag. Serv. Qual. Int. J..

[58] Jovanović L., Kudláček M., Block M.E., Djordjević I. (2014). Self-Efficacy of Pre-service Physical Education Teacher toward Teaching Students with Disabilities in General Physical Education Classes in Serbia. Eur. J. Adapt. Phys. Act..

[59] Özer D., Nalbant S., Aǧlamıș E., Baran F., Kaya Samut P., Aktop A., Hutzler Y. (2013). Physical Education Teachers’ Attitudes towards Children with Intellectual Disability: The Impact of Time in Service, Gender, and Previous Acquaintance. J. Intellect. Disabil. Res..

[60] Teng K.Y., Yeo K.J., Lee C.Y.C., Chin N.S., Balint G., Antala B., Carty C., Aleokol J.-M., Amar I., Kaplánová A. (2021). Primary School Physical Education Teachers’ Efficacy towards Inclusive Education Programme Classrooms in Malaysian Context. Physical Education and Sport for Children and Youth with Special Needs: Researches—Best Practices—Situation.

[61] Aldabas R. (2020). Special Education Teachers’ Perceptions of Their Preparedness to Teach Students with Severe Disabilities in Inclusive Classrooms: A Saudi Arabian Perspective. SAGE Open.

[62] Alahmadi M.A. (2021). Health-Related Physical Fitness Components in Saudi Female Physical Education and Sport Sciences University Students. EC Nutr..

[63] Crawford S. (2011). An Examination of Current Adapted Physical Activity Provision in Primary and Special Schools in Ireland. Eur. Phys. Educ. Rev..

[64] Marron S., Morris S. (2018). Inclusion in Physical Education: Perceptions of Irish and Swiss Student Teachers Following Participation in a European Exchange Programme. Eur. J. Adapt. Phys. Act..

[65] Meegan S., MacPhail A. (2006). Irish Physical Educators’ Attitude toward Teaching Students with Special Educational Needs. Eur. Phys. Educ. Rev..

[66] Önal A., Hergüner G., Uluışık V., Yaman Ç. (2018). Examining the Attitudes of Physical Education Teachers towards Special Education (the Handicapped). Phys. Educ. Stud..

[67] Hwang Y.S., Evans D. (2011). Attitudes towards Inclusion: Gaps between Belief and Practice. Int. J. Spec. Educ..

[68] Schmidt M., Vrhovnik K. (2015). Attitudes of Teachers towards the Inclusion of Children with Special Needs in Primary and Secondary Schools. Hrvat. Rev. Za Rehabil. Istraz..

[69] Tripp A., Rizzo T.L. (2006). Disability Labels Affect Physical Educators. Adapt. Phys. Act. Q..

[70] UNESCO (2015). International Charter of Physical Education, Physical Activity and Sport.

[71] Hutzler Y., Zach S., Gafni O. (2005). Physical Education Students’ Attitudes and Self-Efficacy towards the Participation of Children with Special Needs in Regular Classes. Eur. J. Spec. Needs Edu..

[72] Koh Y. (2017). A Strategy to Improve Pre-service Teachers’ Self-Efficacy Towards Inclusive Physical Education for Students with Intellectual Disability and Autism. Int. J. Inclusive. Educ..

[73] Coates J.K. (2011). Teaching inclusively: Are Secondary Physical Education Student Teachers Sufficiently Prepared to Teach in Inclusive Environments?. Phys. Educ. Sport Pedagog..

[74] Hersman B.L., Hodge S.R. (2010). High School Physical Educators’ Beliefs about Teaching Differently Abled Students in an Urban Public School District. Educ. Urban Soc..

[75] Corona L.L., Christodulu K.V., Rinaldi M.L. (2017). Investigation of School Professionals’ Self-Efficacy for Working with Students with ASD: Impact of Prior Experience, Knowledge, and Training. J. Posit. Behav. Interv..

[76] Alhumaid M.M., Khoo S., Bastos T. (2021). The Effect of an Adapted Physical Activity Intervention Program on Pre-Service Physical Education Teachers’ Self-Efficacy towards Inclusion in Saudi Arabia. Sustainability.

[77] McCambridge J., De Bruin M., Witton J. (2012). The Effects of Demand Characteristics on Research Participant Behaviours in Non-laboratory Settings: A Systematic Review. PLoS ONE.

